# Controversies around the function of LARP1

**DOI:** 10.1080/15476286.2020.1733787

**Published:** 2020-04-01

**Authors:** Andrea J. Berman, Carson C. Thoreen, Zinaida Dedeic, James Chettle, Philippe P. Roux, Sarah P. Blagden

**Affiliations:** a Department of Biological Sciences, University of Pittsburgh, Pittsburgh, USA; b Department of Cellular and Molecular Physiology, Yale School of Medicine, New Haven, USA; c Department of Oncology, University of Oxford, Oxford, UK; d Institute for Research in Immunology and Cancer (IRIC), Université De Montréal, Montreal, Quebec, Canada

**Keywords:** LARP1, La-related protein 1, mRNA, translation, mTOR, mTORC1, cancer, PABP, poly A binding protein, LARP

## Abstract

The RNA-binding protein LARP1 has generated interest in recent years for its role in the mTOR signalling cascade and its regulation of terminal oligopyrimidine (TOP) mRNA translation. Paradoxically, some scientists have shown that LARP1 represses TOP translation while others that LARP1 activates it. Here, we present opinions from four leading scientists in the field to discuss these and other contradictory findings.

## Introduction

Those entering the field of LARP biology may be confused by the literature concerning LARP1. Whilst it is universally agreed that LARP1 is an RNA binding protein (RBP) involved with TOP mRNA translation, scientists have debated its exact function in this process. Some show LARP1 inhibits translation of TOP-containing mRNA transcripts whilst others show LARP1 sustains it, leading to vigorous discussions. In recent years, advances in methods of RNA capture and analysis have revealed a more nuanced and complex picture of LARP1 function. Here we asked four scientists leading LARP1 research teams in the USA, Canada and the UK to summarize their views. Each was asked the following: **Of the LARPs, LARP1 seems the most controversial. How do you reconcile these differing opinions and what evidence have you generated to support its mechanism of action?**

## Philippe Roux (Université de Montréal, Canada)

**Figure 1.**
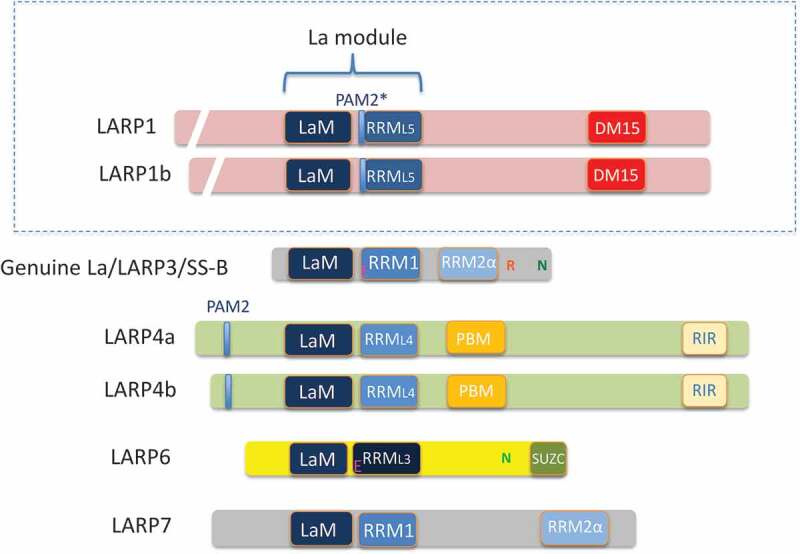
The LARP family members and their conserved domains. Each LARP carries a La motif (**LaM**) adjacent to an RNA recognition motif (**RRM**) that comprises the **La module**, an RNA-binding unit that acts as an independent structural domain. Additional motifs are present on the LARPs that confer them with specific functions. LARP1 and 1B carry a unique C-terminal DM15-repeat containing region (‘**DM15** region’). LARPs 1, 1B, 4A and 4A carry a poly (A) binding protein interaction motif-2 (**PAM-2**) which, in the case of LARP1 and 1b is atypical (PAM2*) and required for PABP binding but LARP1 does not carry the second PABP-interaction motif (**PBM**) present in LARPs 4A and 4B. LARP1 is predominantly located in the cytoplasm but can shuttle into the nucleus. This is despite the fact that no nuclear import (N) or export sequence (E) or a nuclear retention element (R) has yet been annotated in LARP1. Of note, LARP4 and 4b carry a C-terminal Rack1-interacting region (**RIR**) and LARP6 has a **SUC-Z** domain (also called a ‘LaM and S1-associated motif’ or **LSA**). More details on the structural confirmation and binding affinities of these and other LARP motifs can be found in Maraia et al, 2017 [58]. Figure derived from Stavraka and Blagden, 2015 [22] and Maraia et al, 2017[58]

**Figure 2.**
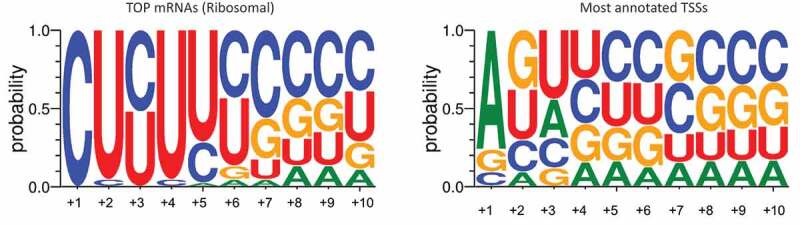
**Characteristic 5ʹTOP sequences**. Members of the 5ʹTOP gene family carry a common cis-regulatory element consisting of an invariant C residue at the Transcriptional Start Site (TSS). In a mature mRNA, this lies immediately after the 7-methylguanosine [m^7^G] cap, represented as m7G-pppC. This is in contrast to non-TOP mRNAs that generally carry a purine at their TSS, represented as m7G-pppG/A. In TOPs, the C residue is followed by a tract of 4–15 pyrimidines (containing a similar proportion of C and U residues) called the ‘TOP motif’ and a GC-rich region lying immediately downstream of the TOP motif. Another feature of *bone fide* 5ʹTOPs is their conservation in orthologues across mammalian species[6]. Here we show sequence logos of the first ten bases of TOP mRNAs encoding ribosomal proteins (left) compared to a representation of the total mRNA population in *Homo Sapiens* (right), using 921 of the most highly conserved transcriptional start sites as reported by Yamashita et al., 2008[59]. Sequence logos were created using Weblogo 3. Although there are only estimated to be 200 TOP genes in the human genome, they comprise 20% total cellular RNA[60]

La-related protein 1 (LARP1) is an evolutionarily conserved RNA-binding protein that belongs to the LARP superfamily [1, 2] (Fig. 1). LARP1 was first characterized in *Drosophila*, where it was shown to bind polyadenylate-binding protein (PABP) and to be required for embryonic development and fertility [3]. More recent studies revealed that LARP1 plays a key role in protein synthesis, as it was shown to regulate both the stability and translation of mRNAs characterized by a 5ʹ terminal oligopyrimidine (5ʹTOP) motif [4,5] (Fig. 2). TOP mRNAs encode proteins involved in the translational apparatus, such as ribosomal proteins and translation factors, but their regulation has remained elusive for decades [6]. The link between LARP1 and TOP mRNAs was confirmed by several groups [7–10], who found that LARP1 associates with the 5ʹ end of TOP mRNAs [7–10]. Interestingly, LARP1 was also shown to associate with the 3ʹ end of TOP mRNAs [4,9], suggesting the interesting possibility that LARP1 may be involved in the circularization of these transcripts. Our results revealed that LARP1 may be an important functional link between the mammalian target of rapamycin (mTOR) pathway and the regulation of TOP mRNAs [5]. LARP1 was initially identified as a potential mTOR complex 1 (mTORC1) substrate [11,12], and subsequent work confirmed that LARP1 binds to Raptor, a component of mTORC1, and is directly phosphorylated by the mTOR kinase [5,7,9]. While these results strongly implicate LARP1 as an important mTORC1 downstream substrate, several questions remain about the precise mechanisms by which LARP1 controls TOP mRNA stability and translation [13].

**Figure 3.**
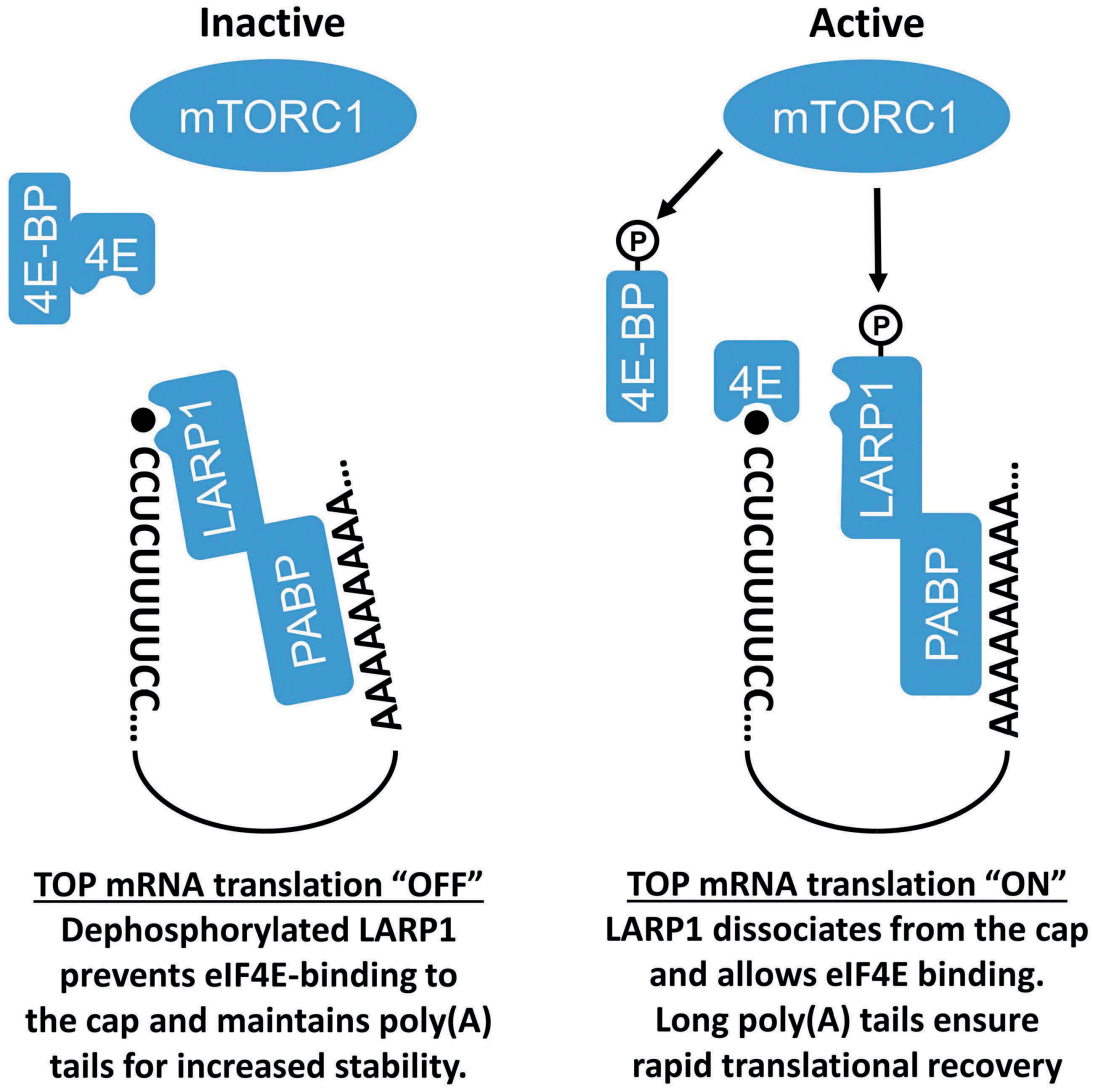
**Model for the role of LARP1 in the regulation of TOP mRNAs**. When mTORC1 is inactive, eIF4F is destabilized via 4E-BPs and dephosphorylated LARP1 binds the 5ʹ ends of TOP mRNAs, repressing their translation and promoting their stability by maintaining the length of poly(A) tails. Upon mTORC1 activation, LARP1 phosphorylation relieves its inhibitory activity, allowing eIF4F to bind TOP mRNAs and resume their translation

LARP1 is thought to interact with mRNAs through several domains, including the RNA-binding La motif (LAM), the RNA Recognition Motif-Like (RRM-L) domain, the PABP-interacting domain, and the highly conserved DM15 region [1,2]. Recent studies have shown that only the DM15 and adjacent region are required for LARP1 to recognize the 5ʹ end of TOP mRNAs and to repress their translation [8,10]. Consistent with this, crystallographic data revealed that the DM15 region specifically binds the 7-methylguanosine 5ʹ-5ʹ triphosphate (m7Gppp) moiety and the invariant first cytidine of TOP mRNAs [8]. In response to mTORC1 inhibition, LARP1 prevents eIF4E-binding to the m7 Gppp cap and blocks eIF4F complex assembly on TOP mRNAs [8]. Interestingly, recent work has shown that LARP1 may act as a phospho-dependent molecular switch that regulates TOP mRNAs in an mTORC1-dependent manner [9]. However, this view has been challenged by several reports showing that translational control of TOP mRNAs is essentially carried out in a Raptor-independent manner [14–16]. In this model (Fig. 3), non-phosphorylated LARP1 binds to the 5ʹ end of TOP mRNAs to repress their translation in the absence of mTORC1 signalling[10]. In response to mTORC1 activation, LARP1 phosphorylation promotes its dissociation from the 5ʹ end of TOP mRNAs, and at the same time, enhances its interaction to the 3ʹ end of TOP mRNAs to facilitate translational recovery following nutrient starvation [9]. Consistent with this possibility, recent work has shown that LARP1 promotes the polyadenylation of TOP mRNAs under conditions where mTORC1 is inhibited [17]. Since a long poly(A) tail typically confers increased stability, it is not surprising that LARP1 depletion was found to reduce TOP mRNA stability [4,7,17], as well as the level of several ribosomal proteins [5,9]. These results suggest that LARP1 may act as a translational repressor when mTORC1 is inactive (via 5ʹ end binding) but may also promote the stability of TOP mRNAs to facilitate translational recovery upon mTORC1 activation (via 3ʹ end binding). The latter may require specific RNA-binding domains within LARP1, and/or its ability to interact with PABP [4,5,7,9].

While more work will be required to clearly define the molecular mechanisms involved in the regulation of TOP mRNAs by LARP1, the net effect of its depletion appears to be reduced cell growth and proliferation [5,9,18]. Consistent with this, LARP1 was found to be significantly upregulated and to correlate with adverse prognosis in several malignancies, including hepatocellular cancer, colorectal cancer, cervical squamous carcinoma, and non-small cell lung cancer [18–21]. These results suggest that elevated LARP1 expression provides a proliferative advantage to cancer cells, which would be consistent with the idea that LARP1 may not solely act as a translational repressor [22]. Interestingly, LARP1 was also suggested to play a role in *5q^−^* myelodysplastic syndrome (MDS), which is a type of anaemia characterized by reduced TOP mRNA levels [23]. The relative proximity of the *LARP1* locus (5q33.2) and the reduction in *LARP1* mRNA levels in *5q^−^* patients suggest that LARP1 may be involved in the pathological development of MDS. Further work will be required to determine if the loss of LARP1 results in impaired TOP mRNA polyadenylation, stability and/or translation in the context of this disease.

Many questions remain about the role of LARP1 in mTORC1-mediated regulation of TOP mRNAs. A better understanding of the molecular details surrounding 5ʹ and 3ʹ end mRNA binding should help decipher its precise involvement in the regulation of these transcripts. Perhaps LARP4A could provide some clues into LARP1 function, as it was also shown to bind and protect poly(A) tails, and thereby promoting mRNA stability and/or translation [24–26]. Interestingly, recent CRISPR-based essentiality screens have shown that cancer cell lines that are dependent on LARP1 are also likely to require LARP4A for proliferation [27]. While these results suggest that LARP1 and LARP4A may have overlapping functions, there is no current evidence that they control the post-transcriptional fate of a shared pool of mRNAs. Consistent with this, LARP4A lacks a DM15 region at its C-terminus [1] (Fig. 1), which likely explains the selectivity of LARP1 towards TOP mRNAs. This important difference suggests that LARP1 may selectively bind to the 5ʹ end of TOP mRNAs via the DM15 region, but also interact with the 3ʹ end of these transcripts via a mechanism that could be shared with LARP4A. A better understanding of the mechanisms by which this occurs, and the potential impact of this interaction on TOP mRNA stability and/or translation should be investigated in the future, as well as how mTORC1 (or another mTOR complex) orchestrates these highly important molecular functions.

## Andrea J. Berman (University of Pittsburgh, USA)

RNA-binding proteins (RBPs) have become a major focus of basic and translational research. RBPs govern cell identity and function through their regulation of RNA metabolism. They not only serve to protect or stabilize associated RNAs, but RBPs direct RNA degradation, translation, and localization. Thus, their dysfunction is often associated with disease. Indeed, our interest in LARP1 was stimulated by Sarah Blagden’s observation that LARP1 protein levels are correlated with ovarian cancer progression. Since this initial observation shared with me at a conference in 2012 and later published, others have established LARP1 as a pivotal node in mTORC1 signalling [5,7,9], solidifying its role in biologically critical pathways that are deregulated in cancer.

Our lab has taken a mechanistic approach to teasing apart the biological roles of LARP1, beginning with analyses of the individual RNA-binding domains or modules. Using a combination of structural biology and biochemistry, we demonstrated the role of the conserved LARP1 DM15 region in the direct binding of the terminal oligopyrimidine (TOP) motif [8,28] characteristic of transcripts encoding the translation apparatus [6]; our structures revealed that the DM15 region of LARP1 directly engages the 7-methylguanosine cap and invariant +1 C of these transcripts, thereby defining LARP1 as a TOP-specific cap-binding protein [8]. Together with Bruno Fonseca, we showed that in binding the cap and TOP motif, LARP1 occludes the binding of translation initiation factors to these transcripts [8]; the association of eIF4G with specific TOP transcripts was probed as a proxy for assessing eIF4F translation initiation complex association. This observation provided a molecular mechanism for the translational repression of these transcripts initially proposed by Bruno Fonseca [7]. Work from Carson Thoreen’s lab confirmed that the integrity of the DM15 region is essential for its ability to repress the translation of TOP transcripts in a cap-dependent manner [10].

**Figure 4.**
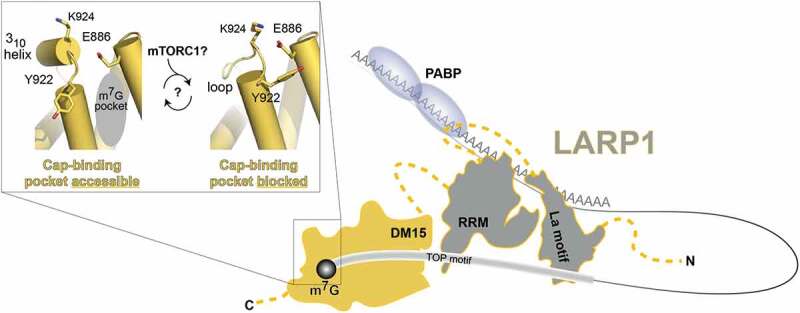
**Model for the contribution of the individual domains to LARP1 RNA-binding activity**. LARP1, shown in yellow and grey with disordered regions shown as yellow dotted lines, binds the 5ʹ 7-methylguanosine cap (m^7^G) of TOP transcripts using the DM15 region. DM15 also binds the first few nucleotides of the TOP motif. The La-module has the ability to bind TOP motif (in a cap-independent manner) and poly(A) RNA simultaneously. The PAM2-like motif most likely interacts with PABP [7]. Inset, one region of the DM15 region exists as a 3_10_ helix or a loop over the course of a 500ns molecular dynamics simulation [28]. In the former conformation, the cap-binding pocket can accommodate the cap; in the latter conformation, Y922 moves into the cap-binding pocket, thereby occluding the cap moiety of a transcript from binding–and possibly inhibiting the DM15 region from binding RNA. Outlines of the La motif and RRM are based on PDBIDs 1s7a and 1s79, respectively, which are solution structures of the domains from genuine La protein determined by the laboratory of Sasi Conte [61]. We do not yet understand how the post-translational modification of the LARP1 DM15 region by mTORC1 regulates these dynamics

In more recent work, we used molecular dynamic simulations to uncover the underlying means by which the DM15 region accommodates the capped-TOP motif in its RNA-binding pocket [29]. Interestingly, amino acids towards the C-terminus of the DM15 region alternate between an unstructured loop and 3_10_ helix. The 3_10_ helix is a secondary structural element that has slightly different parameters than the α-helix; the conformation of these amino acids is directly related to whether the cap-binding pocket is formed and ready to bind the cap of these transcripts. This study also revealed that an amino acid in this loop sequesters the residue in the cap-binding pocket that specifically recognizes the Watson-Crick face of the cap, possibly revealing an important regulatory mechanism for cap binding (Fig. 4).

We have also made significant progress in understanding the role of the LARP1 La-Module in mediating the RNA-binding activity of LARP1. In Al-Ashtal *et al*. (this issue) [30], we demonstrate that the LARP1 La-Module has the ability to simultaneously bind both poly(A) RNA and TOP motif RNA in *in vitro* assays. Importantly, it recognizes these RNAs with similar high affinities (30–40 nM) and in a seemingly ordered manner: the La-Module can bind both RNAs only if it binds poly(A) RNA first, suggesting that the binding of poly(A) RNA might induce a conformational change in the La-Module that opens up a second TOP motif binding pocket. Thus, we predict that one La-Module can bind one TOP motif and one poly(A) oligo simultaneously; however, we cannot exclude the possibility that each of two interacting molecules of the La-Module bind a single RNA in a 2 La-Module: 1 TOP motif: 1 poly(A) complex.

While more work is necessary to further characterize these dynamics, it is important to place these results in the context of the full-length LARP1 protein. As the LARP1 La-Module recognizes the TOP motif in a cap-independent manner [30], it is tempting to speculate that the La-Module and the DM15 region could bind TOP motif simultaneously. Indeed, in this scenario, we would predict that the bound TOP transcript would be translationally repressed (Fig. 3). If the La-Module were also bound to poly(A) RNA and possibly to PABP, as several labs have demonstrated [4,7,31], perhaps the repressed RNA would be circularized [32,33], a conformation that has recently been proposed to occur in stress granules [34]. Consistent with this hypothesis, Jeffrey Chao’s lab has demonstrated a TOP-anchoring role for LARP1 in stress granules and P-bodies [35]; a role for LARP1 in P-bodies was initially proposed by the laboratory of Judith Kimble [36].

On the other side of the mRNA circularization debate is the idea that this conformation is associated with increased translation [37]. Indeed, if the DM15 region of LARP1 releases the cap, perhaps by mTORC1-dependent phosphorylation of residues nearby [38], then the conformation of the LARP1-associated transcript could stimulate its translation, especially if it is already bound to the 40S [23]. This idea is consistent with the role of LARP1 as a translational activator proposed by several labs [5,18,31,39]. Clearly, we do not understand all of the mechanics of this system or contextual trends. The combined expertise of the LARP1 community will allow us to reveal all of the wonderful intricacies of this protein and its role in healthy cells, in cancer [18–20,22,40–41], and in viral infection [42,43].

## Carson C. Thoreen (Yale school of medicine, USA)

Until recently, La-related protein 1 (LARP1) was one of many evolutionarily conserved but functionally nebulous RNA-binding proteins. It belongs to an ancient family of La-related proteins (LARPs) that share a 90 amino acid RNA-binding domain called the La module (LAM) [1]. The original LAM comes from the nuclear protein La, but La and LARPs otherwise share limited homology. Amongst the LARPs, LARP1 also contains an unusual C-terminal RNA-binding domain known as the ‘DM15’ region. Exactly what LARP1 does to RNA has been the subject of increasing (and sometimes contradictory) debate over the last few years. Here, I will describe our interpretation of recent results arguing that LARP1 is a translation repressor and mRNA stabilizer that primarily targets a family of mRNAs called TOP mRNAs.

Unlike other LARPs, LARP1 has been perennially linked to TOP mRNAs. These mRNAs are distinguished by an unbroken series of 5–15 Cs and Us at their 5ʹ terminus known as terminal oligopyrimidine (TOP) motifs [6](Fig. 2). TOP motifs are mostly found on mRNAs encoding translation factors and ribosomal proteins and appear to exist at least throughout vertebrates. Their significance is that they are potent translation regulatory elements. When appended with a TOP motif, the translation of an mRNA becomes exquisitely sensitive to nutrient and other growth-related signals that are transmitted through the mTOR pathway. How and why these essential mRNAs are controlled in this manner has been an ongoing mystery.

Links between LARP1 and TOP mRNAs first surfaced in a study from Natsume and colleagues [4]. LARP1 was found to bind the 3ʹ terminus of the poly-A tail, suggesting it might interfere with deadenylation and thereby stabilize mRNAs. Depletion of LARP1 did indeed deplete levels of several ‘housekeeping’ mRNAs that incidentally belonged to the TOP family, while leaving other abundant mRNAs unperturbed. Several years later, LARP1 emerged again in a proteomic analysis of cap-binding proteins that were isolated using cap-analogue beads [5]. It was concluded that LARP1 doesn’t bind these beads directly, and instead co-purified with other genuine cap-binding complexes (e.g. the eIF4F translation factor). This nonetheless hinted at a translation function for LARP1. Polysome analysis of several TOP mRNAs under normal growth-promoting conditions suggested that LARP1 enhanced their translation. LARP1 therefore seemed to function as a multi-modal positive regulator of TOP mRNA expression by increasing both their stability and translation.

The picture soon became somewhat murkier when Fonseca et al. argued that LARP1 was instead a translation *repressor* [7]. However, a key difference with this study is that LARP1 was also examined under growth-*restrictive* conditions. These are conditions where TOP mRNA translation is normally repressed, and LARP1 was found necessary for the full repression of several representative TOP mRNAs. It nonetheless remained unclear whether LARP1 was directly repressing these transcripts, or instead acting indirectly through the myriad of other translation factors. This uncertainty was underscored by observations that the LARP1 DM15 region selectively bound an RNA mimic of the RPS6 TOP sequence, but with relatively weak affinity (Kd = ~ 500 nM) [29]. It also failed to bind some TOP sequences altogether. Moreover, polypyrimidine sequences are common in mRNAs. A lingering question was how LARP1 would recognize only mRNAs where this sequence was located at the 5ʹ terminus.

The solution to this question has been illuminating. It turns out that the DM15 region of LARP1 doesn’t just recognize polypyrimidine sequences, it also binds the adjacent cap structure. This simple modification increases LARP1 affinity to a Kd of ~20 nM [8]. The significance of this observation, which was made first by Berman and colleagues and soon after by our own group, is two-fold [8,10]. It explains why LARP1 selectively recognizes mRNAs with polypyrimidine sequence at the 5ʹ end (e.g. TOP mRNAs), and also suggests a simple model for how LARP1 might affect both translation and stability. LARP1 binding to the mRNA cap would compete with cap-binding translation factors (e.g. eIF4F), thereby repressing translation. It could also protect TOP mRNAs from destabilization by blocking decapping factors or somehow sequestering them from other decay factors, perhaps in P-bodies where LARP1 may reside [36]. We subsequently showed through a series of functional experiments that both the DM15 region and cap-binding activity were essential for LARP1 to repress TOP mRNA translation [10]. This finding also neatly explains our previous observation that inhibition of eIF4F via the 4E binding protein (4E-BP) translation repressors selectively represses the translation of TOP mRNAs. 4E-BPs, which are active when the mTOR pathway is inhibited, destabilize eIF4F binding to mRNA 5ʹ ends and thereby expose them to LARP1 [44,45].

Our efforts have mostly focused on LARP1’s molecular functions, but its double life as a translation repressor and stabilizer of TOP mRNAs may also explain its biological ambiguity. At first glance, LARP1 would seem to be a potent growth repressor. After all, it represses the translation of TOP mRNAs, which encode proteins that are essential for growth (e.g. ribosomal proteins). However, LARP1 inactivation paradoxically decreases growth rate in at least some tumours [31] and potentially contributes to 5q syndrome, a myelodysplastic disorder with parallels to ribosomopathies caused by loss-of-function mutations in core ribosomal proteins [23]. These outcomes probably reflect decreased TOP mRNA stability rather than increased translation. The net effect of these opposing functions is hard to predict a priori, but LARP1 inactivation could easily *decrease* the overall expression of TOP mRNAs and, consequently, growth. It nonetheless seems likely that LARP1 is important for slowing growth under other circumstances, especially where nutrient and other growth signals vary more rapidly. When and where this occurs remains an interesting and ongoing question.

## Sarah P. Blagden (University of Oxford, UK)

An intriguing feature of LARP1 is its differential regulation in specific cellular contexts. We first characterized the gene in *Drosophila melanogaster* where RNAi depletion of *LARP1* (referred to as *larp*) in embryonic S2 cells caused a reduction in cell proliferation but did not induce cell death [3]. We saw a similar reduction in proliferation in human immortalized non-cancer cell lines after loss of LARP1. This is also consistent with findings by the Thoreen lab that LARP1 knockout in HEK-293T cells resulted in reduced proliferation but did not produce major apoptotic phenotypes [10]. By contrast, in a range of cancer cells, depletion of LARP1 induced apoptosis, particularly in those exposed to cell stress [18,31,39]. This indicates that LARP1 has a pro-survival role in certain cancer cells. Furthermore, these data are consistent with genome-wide CRISPR-Cas9 dependency screens, with the CRISPR (Avana) Public 19Q4 screen identifying 240 of 689 cell lines from a wide range of different cancers as being LARP1 dependent (https://depmap.org/portal/gene/LARP1?tab=overview). This was also in keeping with the clinicopathological data from our team and others showing higher levels of LARP1 protein within tumours was associated with more aggressive cancer growth and worse patient outcome [18–22,31,39].

When we conducted RIP-chip in HeLa cells (a cervical cancer line) we identified over 3000 mRNAs complexed with LARP1 protein [18]. TOP-motif containing mRNAs were represented in this interactome, consistent with the work of others showing them to be LARP1 target genes [4–8]. However, they were by no means the most prominent targets, there was also significant enrichment of oncogenic and cell survival genes [18,39]. In fact, from our HeLa cell-derived LARP1 interactome only 18 of a potential 94 TOP mRNAs (as defined by Meyuhas and Kahan [6]) were identified. This compares with PAR-CLIP data generated by Hong *et al* [9] showing 34/94 and 64/94 TOP mRNAs in the LARP1 interactome under active and repressed mTORC1 signalling, respectively. Despite TOPs being represented in LARP1 interactomes in both HeLa and HEK-293 cells, they accounted for less than 4% of the total mRNAs bound by LARP1 [6,18].

**Figure 5.**
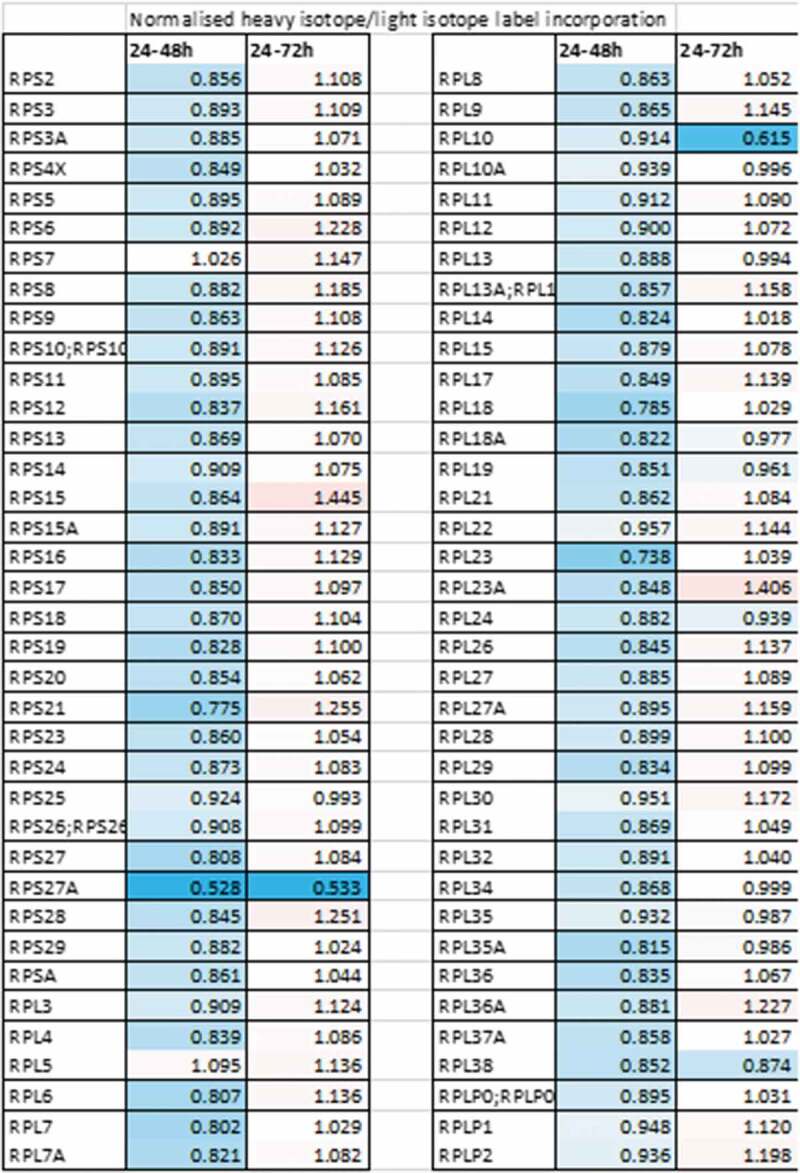
**The impact of LARP1 on protein synthesis**. Pulsed SILAC was performed by replacing unlabelled cell culture medium with labelled medium (R10K8) 24 h after transfection of OVCAR8 cells with an siRNA targeting LARP1 or a non-targeting siRNA. Cells were grown in labelled medium for a further 24 h or 48 h prior to submission of lysates for LC-MS/MS. Levels of protein synthesis of individual proteins following LARP1 knockdown were calculated from the ratios of labelled (newly synthesized) to unlabelled (existing) protein, normalized against the ratio of total nascent protein to existing protein. Downregulated nascent protein synthesis is indicated by values <1 (blue), upregulated nascent protein synthesis is indicated by values >1 (red)

To determine the role of LARP1 in the regulation of non-TOP mRNAs, we selected two antagonistic apoptotic regulators, BIK and BCL2, for further validation. Using luciferase reporter and electromobility shift assays (EMSA) we confirmed a direct interaction between the DM15 region of LARP1 and the 3ʹ UTR of these two mRNAs. Interestingly, whilst LARP1 negatively regulated BIK it stabilized BCL2, with the overall effect of driving cell survival [39]. The mechanism through which LARP1 binding stabilizes some mRNAs but destabilizes others remains elusive. However, given the ability of LARP1 to bind mRNAs with both its La module and DM15 region, and given the binding sites may be present in both untranslated regions and coding sequences of target mRNAs, the mode of LARP1 binding is likely to dictate the mRNA fate [9,30]. More recently, our findings have been substantiated using pulsed Stable Isotope Labelling with Amino acids in Cell culture (SILAC) in ovarian cancer (OVCAR8) cell lines where we observed that LARP1 regulates the *de novo* synthesis of a number of proto-oncogenic and cell survival proteins. These include ribosomal proteins, encoded by TOP mRNAs, which we found were almost universally downregulated in the 24–48 hours after LARP1 knockdown, though levels recovered by 72 hours (Fig. 5). This implies that the effect of LARP1 knockdown is timepoint-dependent, with a compensatory restoration of TOP expression by 3 days. It is likely this restoration is driven by a response to the cellular stress induced by LARP1 depletion. However, the identities of the factors that drive this response have yet to be determined. In a Surface Sensing of Translation (SUnSET) assay we showed LARP1 had an almost exclusively positive impact on global protein synthesis under cellular stress (e.g. induced by cisplatin treatment), moreover the presence of LARP1 was essential for protein synthesis to occur under these conditions. So, unlike those showing LARP1 negatively regulates translation under the control of mTORC1, we have consistently seen the opposite in cancer cells, using methionine uptake [31], SILAC and SUnSET assays [46]. It is unclear why the role of LARP1 should differ in cancer cells versus non-cancer cells, but cancer cells under stress often show aberrant kinase activity, and thus ongoing research is focused on whether post-translational modification of LARP1 induced by stress alters its mode of binding to mRNAs.

**Figure 6.**
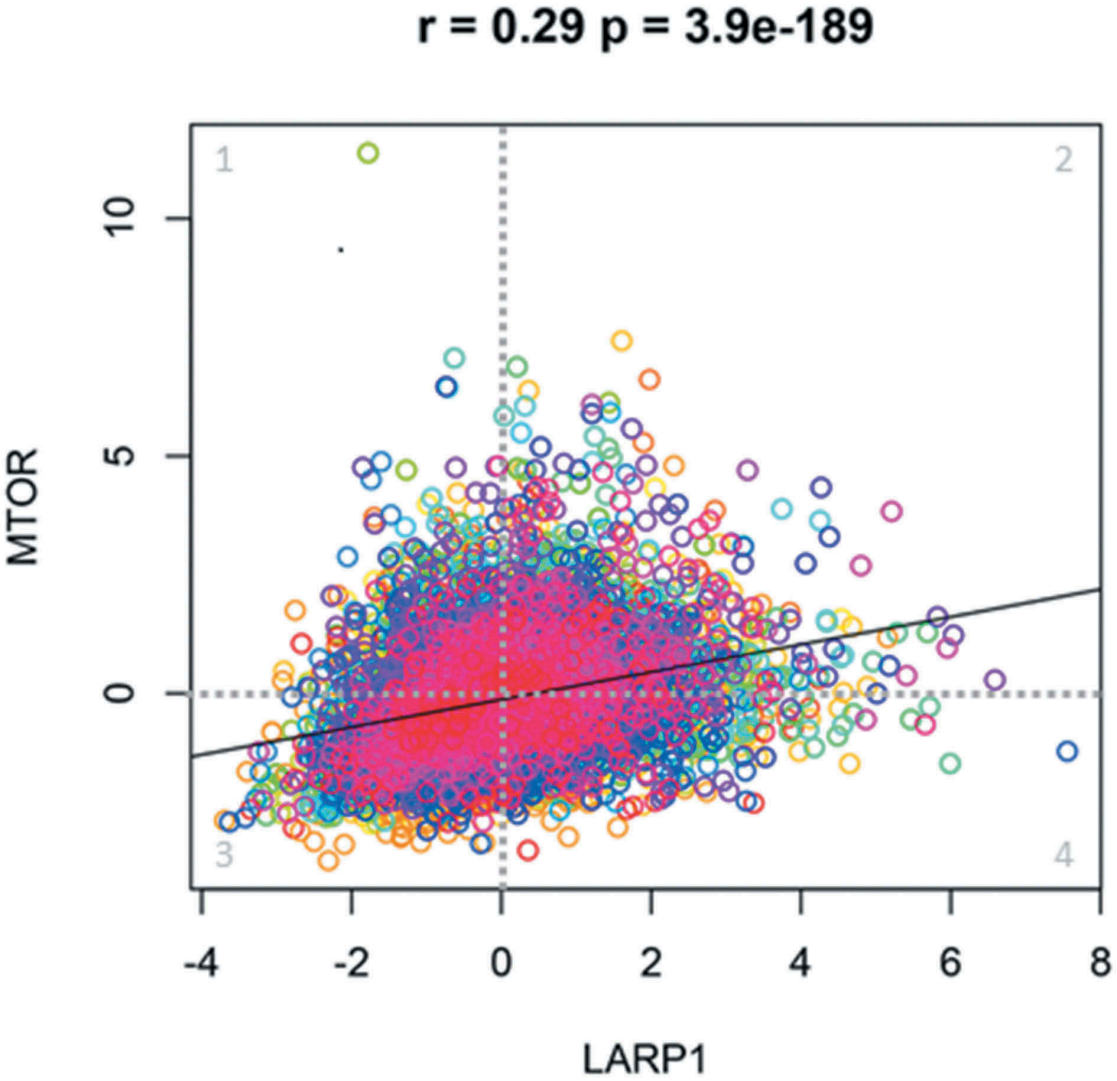
**Correlation between LARP1 and mTOR expression**. Scatter plot showing there is a weakly positive correlation in the Z-score of mRNA expression between LARP1 (X axis) and mTOR (Y axis) in 32 different cancer sets publicly available from TCGA PanCancer Atlas. Each data point represents an individual patient and each colour a different cancer type. Quadrants 1–4 show patients with 1) high mTOR and low LARP1 (1755 patients), 2) high mTOR and high LARP1 (2265 patients), 3) low mTOR and low LARP1 (3889 patients), and 4) low mTOR and high LARP1 (2125 patients). Z-score of mRNA expression, Pearson’s correlation coefficient r = 0.29 with significance value p = 3.89e-189. Data was analysed using cBioPortal [62,63]

Furthermore, we have seen differences in the mTORC1-LARP1 axis in cancer cells compared with non-malignant cells. Using HEK293T cells derived from a non-cancerous embryonic kidney tissue, Bruno Fonseca showed a clear interaction between LARP1 and the mTORC1 complex component RAPTOR [13]. In our lab, using various ovarian and other cancer cell lines, we have been unable to demonstrate an association between LARP1 and RAPTOR under any conditions tested. These co-immunoprecipitation assays were performed with and without cisplatin treatment in multiple independent experiments and subsequently repeated using different anti-LARP1 antibodies [31,46]. This suggests that in certain cancer cells at least, under both non-stressed and stressed conditions, LARP1 is not in complex with RAPTOR. Intriguingly, we have previously shown that LARP1 binds and stabilizes mTOR mRNA [18], suggesting LARP1 acts upstream of mTOR in this context. Exposure to certain cellular stresses such as nutrient deprivation, hypoxia, oxidative and genotoxic stress causes TSC1/2-mediated inactivation of mTORC1 and attenuation of cap-dependent translation [47,48] but under these conditions LARP1 continues to drive protein synthesis and cell survival [39]. When we correlated the expression levels of mTOR and LARP1 in a series of cancer cases, there was a weakly positive correlation between the two (r = 0.29) indicating LARP1 and mTOR often function independently of each other (Fig. 6). This points to an mTORC1-independent regulation of LARP1 for the activation of protein synthesis under certain conditions. Using tandem mass spectroscopy, we identified multiple putative phosphorylation sites along LARP1 protein (at T138, S517, S521, S526, S546, S567, S766, S774 and S830) in addition to the serines at S766 and S774 which have been postulated as possible mTORC1 phospho-sites [11,38]. Other phosphorylation sites within LARP1 have been identified following mitotic arrest or exposure to DNA damage stress [49,50]. It is therefore likely that LARP1 is phospho-regulated by proteins other than mTORC1 under different cellular contexts and when mTORC1 is inhibited. Several other kinases that phosphorylate LARP1 have already been identified, including CDK1 and AKT, which may phosphorylate LARP1 to promote biosynthesis of the translation machinery, and PINK1 which, in *Drosophila* oocytes, phosphorylates LARP1 during mitochondrial biogenesis [51,52–57]. These kinases are known to be activated during cellular stress, supporting the hypothesis that LARP1 may promote altered gene expression and cell survival under stress conditions.

As noted, in addition to TOP mRNAs, LARP1 binds thousands of non-TOP mRNAs [9,18]. Unlike the TOPs, these mRNAs display LARP1 binding sites in all regions of the molecule, including the 5ʹ and 3ʹ-UTRs and the coding region. Efforts to identify common sequence elements or secondary structural motifs among these non-TOP mRNAs have thus far been unsuccessful. The domains of LARP1 involved in their binding are poorly characterized as are the drivers of these interactions. This is a crucial area of research required to further understand the function of LARP1.

Here we speculate on a model whereby LARP1 acts as an mTORC1-dependent regulator of TOP mRNA translation in non-malignant cells but as a constitutive activator of TOP and pro-survival mRNA translation in the cancer cells in which it is upregulated (Fig. 8). Thus, in normal cells, when mTORC1 is inactive under nutrient-poor conditions, the DM15 region binds the 5ʹ TOP sequence to repress translation, while other regions of LARP1 (such as the La module [30]) also contact the mRNA to promote its stabilization. When nutrients are restored, mTORC1 phosphorylates LARP1, releasing the repressive DM15 binding but maintaining the binding of the La module to the mRNA to promote its stabilization and translation. Consistent with this model, previous studies have shown that in mTOR-inactive conditions, LARP1 acts as a translational repressor and protects 5ʹ TOP mRNAs [7,10]. Cancers often show aberrant mTORC1 activity, with constitutive activation but also inactivation being reported. In LARP1-dependent cancer cells, LARP1 is phosphorylated by mTOR or by other pro-mitogenic kinases (such as CDK1 or AKT) and thus LARP1-mediated translational activation may be mTOR-dependent or independent. A possible model is that in the absence of mTORC1, and upon phosphorylation of LARP1 by upstream kinases, the DM15 releases the 5ʹ cap of the TOP mRNA but La module binding is sustained. In certain circumstances, the DM15 may bind to other (non-5ʹcap) regions of the mRNA, such as the coding region or 3ʹUTR, following its release from the 5ʹ cap. Even in this circumstance, LARP1 appears to have a dual effect; stabilizing some mRNAs whilst destabilizing others but, overall, has a positive effect on protein synthesis. The identification of LARP1 in stress granules and P-bodies indicates it is capable of shuttling its target mRNAs to specific subcellular localizations that determine their fate. This is the subject of ongoing research.

## Conclusions

The consistent presence of LARP1 in the genome of organisms for an estimated 2 billion years implies it has a fundamental role in survival [58]. This is supported by data obtained over the last decade positioning LARP1 within the mTOR signalling cascade. The principal function of mTORC1 is to regulate cellular homoeostasis in response to nutrient supply. When nutrients are plentiful, mTORC1 activates cell growth via cap-dependent translation and synthesizes the cellular ‘hardware’ (ribosomes and translation factors that are encoded by TOP-bearing mRNAs) to support anabolism and drive proliferation, a process termed ‘ribosome biogenesis’. As LARP1 is phospho-regulated by mTORC1 and has a propensity to bind TOP mRNAs, it suggests LARP1 is principally tasked with the biogenesis arm of the mTOR axis.

**Figure 7.**
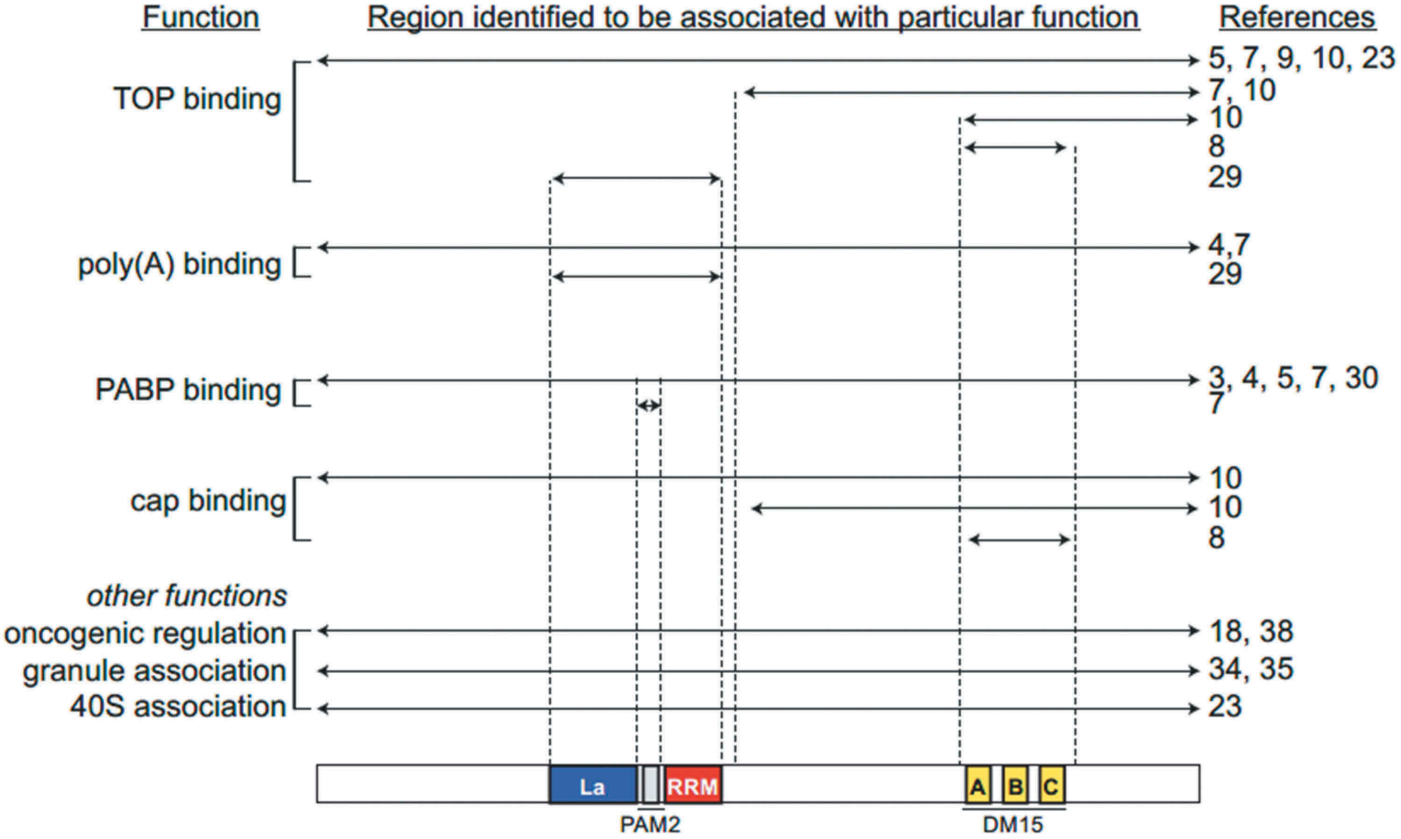
**A summary of the literature in the context of identified LARP1 functions**. Functions are listed to the left with the associated references on the right. In the centre, the construct of LARP1 for which that function was identified in the indicated references is shown aligned with the domain organization cartoon at the bottom. For example, studies that associated the full-length LARP1 with a function are listed beside an arrow extending from N- to C- terminus. Studies that honed in on smaller regions of the protein, or that used smaller constructs, are indicated with the appropriate arrows

**Figure 8.**
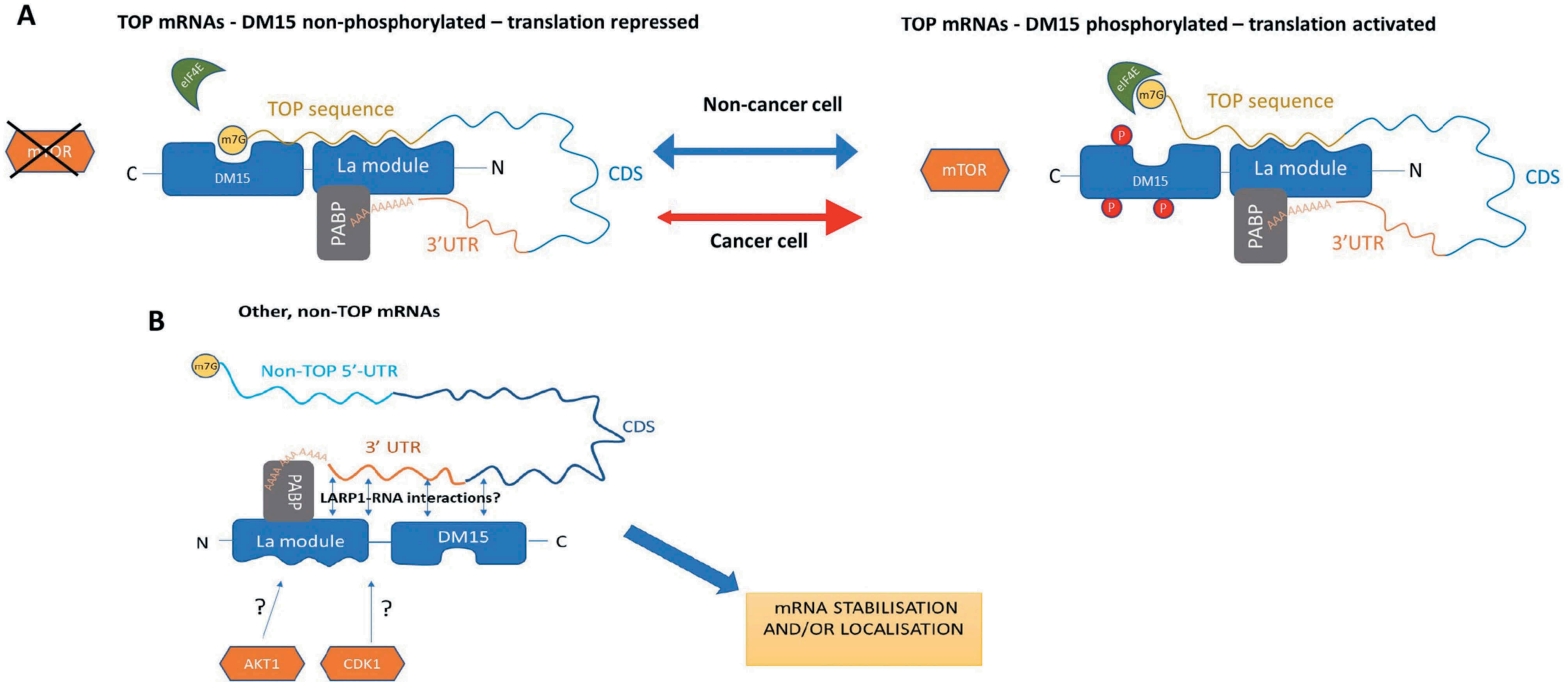
**Unifying model of the regulation of TOP and non-TOP mRNAs by LARP1**. **(A) Regulation of TOP mRNAs in the mTORC1 inactive condition**. DM15 is unphosphorylated and outcompetes eIF4E to bind the 5ʹ cap. mRNA is stabilized but translation is repressed due to lack of eIF4E binding. Phosphorylation of the DM15 by mTOR (and possibly other kinases) causes loss of repressive DM15 binding to the 5ʹ cap and activation of translation. In this model, binding by the La module and interaction with PABP results in mRNA stabilization, while DM15 release allows active translation. It is likely that LARP1 switches between the two phosphorylation states according to nutrient availability and mTOR activation. In cancer cells one or more kinases that phosphorylate LARP1 (including mTOR) may be constitutively active, resulting in loss of DM15 repressive binding. Phosphorylation sites on other regions of LARP1 aside from DM15 are known to exist but have yet been characterized (not shown). **B) Regulation of non-TOP mRNAs**. LARP1 can act as an oncoprotein even when mTOR is inactive, indicating that other kinases (such as CDK1 and AKT1) may phosphorylate LARP1 (at regions yet to be defined) and activate translation. Multiple non-TOP mRNA targets are bound by LARP1 [9,18,23]. CLIP data suggests binding is mainly across the CDS and 3ʹ UTR but can also occur in the 5ʹ UTR of target genes. The regions of LARP1 responsible for these interactions (La module, DM15 or both) have not been conclusively demonstrated. It is likely that the exact mode of binding may determine mRNA fate

In this review, four scientists were asked to reconcile some seemingly contradictory findings as to whether LARP1 is a TOP activator or repressor. They agreed that LARP1 is a *bona fide* regulator of TOP mRNAs, confirmed by various structural studies demonstrating the specificity of LARP1 (and in particular the DM15 region) towards TOP mRNAs. The domains required for these specific interactions and functions are summarized in Fig. 7. In developing this review, the authors came to an intriguing conclusion: LARP1 exhibits distinct mechanisms for binding TOP and non-TOP mRNAs that may be perturbed in cancer cells (Fig. 8). In this model, in the mTORC1 ‘off’ or growth-restrictive environment, the DM15 region primarily mediates translation repression by competing with eIF4E for the 5ʹ cap of TOP mRNAs whilst the La module stabilizes the repressed transcript. In the mTORC1 ‘on’ or nutrient rich context, the DM15 is phosphorylated and therefore disassociates from the 5ʹ cap but the La module remains in contact with the mRNA, circularizing and stabilizing it to facilitate translation. This implies that LARP1 is a cellular gas pedal, altering its binding configurations to increase or decrease the stability of TOP mRNAs and thus modulating ribosome biogenesis under mTORC1’s command. When nutrients are plentiful and mTORC1 is active, LARP1 uses the La module to clasp and stabilize TOP mRNAs. Conversely, when nutrient supply is low and mTOR signalling is extinguished, LARP1 uses the DM15 region to bind and repress TOP transcripts. This gives an intriguing insight into the ability of LARP1, through alterations in its structural conformation, to act as a positive or negative mTORC1 effector protein.

We have consistently observed that cancer cells with high levels of LARP1 have strong dependence on it for survival. Abrupt loss of LARP1 in these cells causes profound apoptosis, implying the cells are ‘addicted’ to LARP1. This is in keeping with clinical data showing elevated levels of LARP1 protein in tumour cells correlate with poorer patient survival and (in-vivo) lower sensitivity to chemotherapy. In addition to its role in regulating TOP mRNAs, LARP1 also binds and regulates thousands of non-TOP mRNAs encoding cell survival, metabolism and stress-response proteins [9,18]. Although some cancers co-express high levels of LARP1 and mTOR, for the majority of cancers they are uncoupled (Fig. 6). Here other pro-mitogenic kinases may replace mTOR as drivers of LARP1 [9,51,52]. The DM15 region of LARP1 has been to shown to bind the UTRs of multiple non-TOP mRNA targets in an mTOR-independent manner [18,39] (Martin Bushell, by communication). In such cases, the DM15 of LARP1 may remain constitutively phosphorylated, leaving the ‘cellular gas pedal’ fully active and binding a wider interactome than TOPs alone. The role of the La module in interacting with non-TOP mRNAs remains unclear, although its interaction with PABP is likely to stabilize binding. In the model shown in Fig. 8B, LARP1 contacts the 3ʹ UTR or CDS of non-TOP mRNAs, via either its DM15 region or La module. This interaction, perhaps along with ongoing engagement between the La Module and PABP, stabilizes the mRNA. It is also possible, reflecting Andrea Berman’s recent findings, that the La module could bind and protect the 5ʹ UTR in the absence of the DM15 binding.

As LARP1 is known to play a crucial role in permitting continued translation and cancer cell survival under stress, it is possible that this ‘non-TOP role’ of LARP1 is driven by stress conditions. Here, the cancer cell is exquisitely dependent on LARP1 for the translation of essential oncogenic proteins when cap-mediated translation is repressed. Critical future work will be to clarify the binding patterns and the pathways regulating LARP1 in these different contexts. While research has been focused on the interactions between LARP1 and mRNAs, it is also likely that its protein interactions (including to its partner PABP) are significant, perhaps for the subcellular localization of LARP1 as well as its ability to select and interact with these mRNA targets. As nutrient-dependent phospho-regulation by mTORC1 is perhaps the default role for LARP1, an understanding of its switch to stress-regulated survival signalling is the key to unlocking its function in cancer and other diseases. The highly collaborative relationship between scientists in this field, as exemplified by this article, creates the optimal environment for these discoveries to be made.
